# 2020 ESC Guidelines on acute coronary syndrome without ST-segment elevation: Recommendations and critical appraisal from the Dutch ACS and Interventional Cardiology working groups

**DOI:** 10.1007/s12471-021-01593-4

**Published:** 2021-07-07

**Authors:** F. Arslan, P. Damman, B. Zwart, Y. Appelman, M. Voskuil, A. de Vos, N. van Royen, J. W. Jukema, R. Waalewijn, R. S. Hermanides, P. Woudstra, T. ten Cate, J. S. Lemkes, M. A. Vink, W. Balder, M. L. J. van der Wielen, P. J. Vlaar, D. J. van der Heijden, S. Assa, A. W. van ’t Hof, J. M. ten Berg

**Affiliations:** 1 Vivantes Klinikum am Urban, Berlin, Germany; 2 St. Antonius Hospital, Nieuwegein, The Netherlands; 3 Radboud University Medical Center, Nijmegen, The Netherlands; 4 Elisabeth-TweeSteden Hospital, Tilburg, The Netherlands; 5 Amsterdam University Medical Centers, Amsterdam, The Netherlands; 6 University Medical Center Utrecht, Utrecht, The Netherlands; 7 Catharina Hospital, Eindhoven, The Netherlands; 8 Leiden University Medical Center, Leiden, The Netherlands; 9 Gelre Hospital, Apeldoorn, The Netherlands; 10 Isala Hospital, Zwolle, The Netherlands; 11 Medisch Centrum Leeuwarden, Leeuwarden, The Netherlands; 12 Onze Lieve Vrouwe Gasthuis, Amsterdam, The Netherlands; 13 Treant Zorggroep, Hoogeveen, The Netherlands; 14 Haaglanden Medical Centre, The Hague, The Netherlands; 15 University Medical Center Groningen, Groningen, The Netherlands; 16 Maastricht University Medical Center, Maastricht, The Netherlands

**Keywords:** NSTE-ACS, NSTEMI, Dual antiplatelet therapy, Invasive management

## Abstract

Recently, the European Society of Cardiology (ESC) has updated its guidelines for the management of patients with acute coronary syndrome (ACS) without ST-segment elevation. The current consensus document of the Dutch ACS working group and the Working Group of Interventional Cardiology of the Netherlands Society of Cardiology aims to put the 2020 ESC Guidelines into the Dutch perspective and to provide practical recommendations for Dutch cardiologists, focusing on antiplatelet therapy, risk assessment and criteria for invasive strategy.

## Introduction

Acute coronary syndrome without ST-segment elevation (NSTE-ACS) is a challenging field to manage within the spectrum of coronary artery disease, given its diversity in diagnostic and treatment strategies. To provide the best available care for NSTE-ACS patients, it is vital to have knowledge of the pathophysiology, clinical presentation, diagnostic criteria and risks and benefits of the proposed therapy (both medical and invasive) and to know how regional healthcare systems are organised [1].

In its 2020 Guidelines for the management of NSTE-ACS, the European Society of Cardiology (ESC) has altered several recommendations, with a great impact on the treatment of NSTE-ACS [2]. It is without question that these alterations were made with great care for the better good of our patients and were based on new insights substantiated with data from clinical trials and observations. Nevertheless, given the impact of the guidelines, critical appraisal is of utmost importance to put these recommendations into perspective. In addition, recommendations are formulated in a certain manner to provide guidance for the majority of physicians and nations, while regional differences in the way healthcare is organised and the availability of technology may influence the relevance and significance of the recommendations.

The Dutch ACS working group and the Working Group of Interventional Cardiology of the Netherlands Society of Cardiology (*NVVC*) have received many questions and requests from Dutch cardiologists on how to interpret the 2020 ESC Guidelines on NSTE-ACS and how to implement them in daily practice. Furthermore, multiple recommendations in the guidelines have a large logistical impact when translated to the Dutch setting. Therefore, the Dutch ACS working group has published multiple consensus documents on recent ACS guidelines to assist in the critical appraisal in the Dutch clinical setting.

**Fig. 1:**
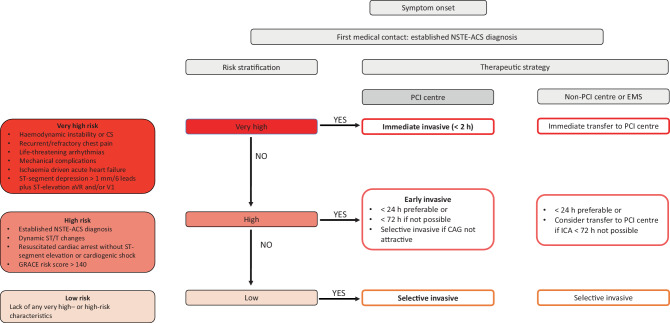
Proposed timing and strategy for acute coronary syndrome without ST-segment elevation (*NSTE-ACS*) in the Dutch setting. *EMS* emergency medical services, *CS* cardiogenic shock, *PCI* percutaneous coronary intervention, *GRACE* Global Registry of Acute Coronary Events, *CAG* coronary angiography, *ICA* invasive coronary angiography

In the current consensus document, the working groups together provide a schematic overview of the 2020 recommendations on those themes that were regarded as most debatable and relevant for the situation in the Netherlands (Fig. 1 and Tab. 1).

**Table 1:** Differences between 2020 European Society of Cardiology (*ESC*) guidelines for acute coronary syndrome without ST-segment elevation *(NSTE-ACS*) and Dutch ACS and Interventional Cardiology working groups consensus paper

2020 ESC Guidelines	Dutch working groups consensus
– Routine P2Y_12_ pretreatment is not recommended when coronary anatomy is unknown and early invasive strategy (< 24 h) is planned – Pretreatment may be considered in patients who cannot undergo early invasive strategy	– Routine P2Y_12_ pretreatment is not recommended when coronary anatomy is unknown and early invasive strategy (< 24 h) is planned – Pretreatment may be considered in patients who cannot undergo early invasive strategy
– Prasugrel should be considered in preference to ticagrelor for NSTE-ACS patients who proceed to PCI	– Use of prasugrel or ticagrelor is recommended for patients who proceed to PCI
– DAPT with prasugrel or ticagrelor is recommended standard for NSTE-ACS	– Use of prasugrel or ticagrelor is recommended for patients who proceed to PCI
	– Clopidogrel is recommended for patients > 70 years
	– DAPT with ticagrelor or clopidogrel (age > 70 years or high-risk bleeding patients) is recommended for patients who are medically treated for NSTE-ACS
– Early (< 24 h) invasive strategy and same-day transfer to PCI centre is recommended in patients with established NSTE-ACS diagnosis or with 1 high-risk criterion^a^	– Early invasive strategy is logistically preferable in patients with GRACE risk score > 140
	– Delayed invasive strategy (< 72 h) is a safe and justifiable alternative for early invasive strategy
	– If performing ICA within 72 h is not possible, consider transfer to PCI centre
– ICA < 24 h is recommended in resuscitated patients with NSTE-ACS	– It is justified to await neurological recovery in resuscitated NSTE-ACS patients

*PCI* percutaneous coronary intervention, *DAPT* dual antiplatelet therapy, *ICA* invasive coronary angiography, *GRACE* Global Registry of Acute Coronary Events

^a^ See Fig. 1

## Pretreatment with P2Y_12_ receptor inhibitors

Based on the results of the recently published ISAR-REACT 5 trial and the observations from the Swedish Coronary Angiography and Angioplasty Registry (SCAAR) database, the current guidelines give a Class I, level of evidence (LoE) B recommendation for treatment with prasugrel or ticagrelor in NSTE-ACS, with a preference for prasugrel (Class IIa, LoE B). In cases where both prasugrel and ticagrelor are not available, contraindicated or not tolerated, clopidogrel is recommended (Class I, LoE C). In addition, routine pretreatment with any type of P2Y_12_ receptor inhibitor is no longer recommended (Class III, LoE A). The 2015 ESC Guidelines could not make any recommendations on this topic; however, pretreatment with a P2Y_12_ receptor inhibitor (preferably ticagrelor) in conservatively managed patients was recommended.

### ISAR-REACT 5 trial

The 2020 ESC Guideline recommendations are based on the ISAR-REACT 5 trial, which was a comparison of two distinct treatment strategies involving two different drugs [3]. Patients with ACS, including ST-elevation myocardial infarction (STEMI), planned for invasive treatment were randomised to ticagrelor or prasugrel. Patients who were assigned to ticagrelor received the loading dose as soon as possible after randomisation. In the prasugrel group, timing of the initiation of the drug treatment depended on the clinical presentation. In patients with STEMI, prasugrel was administered as soon as possible after randomisation. In patients with NSTE-ACS, the loading dose of prasugrel was postponed until the coronary anatomy was known and before proceeding to percutaneous coronary intervention (PCI). In patients with coronary angiography-confirmed ACS but who were treated conservatively, prasugrel was recommended. Coronary angiography in the NSTE-ACS group was performed within a mean 4 hours after randomisation (personal communication with investigators from the ISAR group).

Although more patients in the ticagrelor group received the study drug initially, at discharge roughly 81% in both groups (STEMI vs non-STEMI (NSTEMI)/unstable angina) were on the assigned P2Y_12_ receptor inhibitor. Numbers of patients on the study drug in the NSTEMI population were not reported. At 1 year, the ticagrelor-based strategy showed a significantly higher incidence in the composite endpoint of death, MI or stroke (hazard ratio (HR) 1.36, 95% confidence interval (CI) 1.09–1.70, *p* = 0.006), primarily driven by higher rates of MI. The incidence of major bleeding (Bleeding Academic Research Consortium (BARC) type 3–5) did not differ between the strategies.

The ISAR-REACT 5 trial is a landmark trial and the largest randomised controlled trial (RCT) comparing prasugrel with ticagrelor. However, several comments are to be made on the trial design and results that are relevant for the interpretation of the data. First, instead of a direct comparison between prasugrel and ticagrelor, the comparison is made between treatment strategies including preloading with ticagrelor and no preloading with prasugrel.

Second, approximately 19% of the patients in each strategy group did not receive the randomly assigned trial drug. Of the remaining population, a significant number of patients discontinued study medication: 12.5% in the prasugrel and 15.2% in the ticagrelor strategy group. Such high numbers of discontinuation may have had a major impact on the trial results.

Third, from a pathophysiological view, it is difficult to understand why the ticagrelor treatment led to more ischaemic events when it was given earlier and to more patients than prasugrel. The CHAMPION PHOENIX trial showed that early and potent P2Y_12_ receptor inhibition results in a significant reduction of stent thrombosis after an intravenous bolus and infusion of cangrelor compared with an oral loading dose of clopidogrel (0.8% vs 1.4%, *p* = 0.01).[4] The mechanism behind the observed reduction is the swift P2Y_12_ receptor inhibition with cangrelor, thereby supporting a ‘the sooner, the better’ approach in P2Y_12_ receptor inhibition. However, this was not the case in the ISAR REACT 5 trial.

### SCAAR database

In the recent report from the SCAAR database, 64,857 patients were included for analysis. Of these patients, 59,894 received pretreatment consisting mainly of clopidogrel or ticagrelor (1.8% received prasugrel) and 4963 were not pretreated with P2Y_12_ receptor inhibitors [5]. The analysis showed that pretreatment did not improve survival at 1 month and 1 year but was associated with more in-hospital bleedings (6.0% and 7.5%, respectively, *p* = 0.02). However, adjusted for age and sex, 30-day mortality was significantly lower with pretreatment (1.4% vs 2.5%, *p* < 0.001). A policy change in one Swedish region (Västra Götaland County) dictated that pretreatment was not recommended per protocol after 2016. A comparison of the data before and after the policy change showed the same results, albeit a less impressive reduction in in-hospital bleedings (8.5% vs 8.1%, *p* = 0.006).

Several limitations to the analysis hamper the interpretation of the data. The context is an observational analysis of, nevertheless, an impressive amount of data. Confounding factors and bias are serious concerns despite the fact that the analyses were performed after adjustments for a high number of variables. Furthermore, radial access was significantly lower in patients with pretreatment and could have had a major impact on bleeding events.

### Choice of P2Y_12_ receptor inhibitor

With regards to choosing between prasugrel and ticagrelor, the ISAR-REACT 5 trial hints at superiority of prasugrel. However, this is the only large RCT comparing prasugrel and ticagrelor, while in a recent meta-analysis of 12 trials, including all three P2Y_12_ receptor inhibitors, only ticagrelor was associated with a mortality benefit when compared with clopidogrel [6]. In addition, the recent POPular Age trial showed that patients with NSTE-ACS > 70 years of age who are treated with clopidogrel experience significantly fewer bleeding complications without an increased thrombotic risk compared with ticagrelor or prasugrel [7]. Hence, we support the routine use of prasugrel or ticagrelor and recommend clopidogrel in patients > 70 years of age. Factors to be taken into account in choosing either P2Y_12_ receptor inhibitor include logistical and patient- and drug-related considerations (one P2Y_12_ receptor inhibitor for all indications, dosing frequency, side effects, prasugrel contraindicated in stroke patients and no net benefit in elderly).

### Summary

The working groups recognise the recent data on pretreatment and the choice of P2Y_12_ receptor inhibitors. Despite the noted limitations of the ISAR-REACT 5 trial and the recent SCAAR database analysis, we do not recommend routine P2Y_12_ receptor inhibition pretreatment in NSTE-ACS (in line with the 2020 ESC Guidelines) when invasive coronary angiography (ICA) is expected the same day (Fig. 1). However, for patients with established NSTE-ACS who are not scheduled for ICA on the same day but later during the index hospitalisation, the working groups recommend to consider pretreatment with ticagrelor, or clopidogrel in patients > 70 years of age or in patients with a high bleeding risk (Academic Research Consortium for High Bleeding Risk ≥ 1 major or ≥ 2 minor criteria (Tab. 7 of the 2020 ESC Guidelines on NSTE-ACS), or Predicting Bleeding Complication in Patients Undergoing Stent Implantation and Subsequent Dual Antiplatelet Therapy (PRECISE-DAPT) score ≥ 25).

## Duration of antiplatelet therapy

The choice and duration of a specific P2Y_12_ receptor inhibitor in combination with aspirin (dual antiplatelet therapy (DAPT)) or anticoagulation (dual antithrombotic therapy) should reflect a tailor-made decision in which the ischaemic risk and bleeding risk are weighed. The default strategy in patients with NSTE-ACS is 12 months DAPT (Class I, LoE A recommendation).

In patients with a high risk of bleeding, clopidogrel is the preferred P2Y_12_ receptor inhibitor choice and the DAPT duration can be shortened to 3 months. DAPT (aspirin plus clopidogrel) with a duration of 1 month is recommended for patients with a very high risk of bleeding, defined as having had a bleeding in the past month and/or scheduled for non-deferrable surgery. After the first month, clopidogrel monotherapy is advised.

Recent trials have investigated P2Y_12_ monotherapy as a new antiplatelet strategy after ACS. The TWILIGHT trial examined ticagrelor monotherapy after 3 months of DAPT versus 12 months of DAPT in patients at high risk of bleeding or ischaemic events who had undergone PCI [8]. Over two-thirds of the patients had ACS. The primary endpoint of BARC major bleeding was significantly reduced by omitting aspirin. The trial was not powered for ischaemic outcomes. The TICO trial, in which 3056 patients with NSTE-ACS were randomised, also showed a significant reduction in major bleeding (1.7% vs 3.0%, *p* = 0.02) and cardiovascular events (3.9% vs 5.9%, *p* = 0.01) in patients who switched to ticagrelor monotherapy after 3 months of DAPT [9]. Until further evidence of ischaemic outcomes in patients at high ischaemic risk is available, a Class IIa, LoE A recommendation is given for this strategy.

In patients taking an oral anticoagulant (OAC) or a non-vitamin K antagonist OAC (NOAC), DAPT is not recommended before ICA. In patients undergoing PCI, a short period (< 1 week) of triple antithrombotic therapy with aspirin, a P2Y_12_ receptor inhibitor (preferably clopidogrel) and a NOAC is recommended. The guidelines recommend to continue dual antithrombotic therapy for 12 months (Class I, LoE A). In patients at high risk of bleeding, dual antithrombotic therapy is recommended for 6 months (Class IIa, LoE B). In patients with a high ischaemic risk (Table 11 of the 2020 ESC Guidelines on NSTE-ACS) for whom anatomical or procedural and/or clinical characteristics outweigh the bleeding risk (e.g. excessive stent length used in a diabetic patient with peripheral artery disease), triple antithrombotic therapy duration may be prolonged up to 1 month. The above-mentioned strategies, with a preference for NOAC over vitamin K antagonists, are summarised in Figures 7 and 8 of the 2020 ESC Guidelines on NSTE-ACS and endorsed by the working groups [2].

## Medical treatments

In medically managed NSTE-ACS, the benefits of ticagrelor over clopidogrel were consistent in a substudy of the PLATO trial.[10] Prasugrel was not superior to clopidogrel in medically managed NSTE-ACS in the TRILOGY ACS trial.[11] The working groups therefore recommend ticagrelor (in low-risk bleeding patients) or clopidogrel (in high-risk bleeding patients) for those who are treated medically for NSTE-ACS. In line with the 2020 ESC Guidelines, we endorse the use of a single antiplatelet agent in combination with an OAC or a NOAC for at least 6 months in patients with a need for oral anticoagulation who are treated medically for NSTE-ACS.

## Invasive strategies

ICA can be performed routinely or selectively. With a selective invasive strategy, ICA is only performed after recurrent symptoms, or based on evidence of inducible ischaemia or coronary obstruction using non-invasive (imaging) modalities. It has been clearly demonstrated in several recent meta-analyses that a *routine* invasive strategy does not reduce (all-cause) mortality or ischaemic events when compared with a selective invasive strategy, but that it is associated with an increased risk of periprocedural MI and bleeding [12–14]. On the other hand, a shorter ischaemic period and shorter hospitalisation have been observed in subgroups [12–14]. In the 2020 ESC Guidelines on NSTE-ACS, there is a tendency to perform angiography earlier. We will discuss these recommendations below.

### Immediate (< 2 h)

We endorse the recommendations in the 2020 ESC Guidelines that immediate (analogous to STEMI management) invasive strategy is necessary in very high-risk patients, given the poor prognosis if left untreated. The presence of ≥ 1 very high-risk criterion justifies immediate ICA or transfer to a PCI centre (Fig. 1).

### Early (< 24 h)

The 2020 ESC Guidelines give a Class I, LoE A recommendation for ICA within 24 h in the presence of an established NSTE-ACS diagnosis or > 1 high-risk criterion (Fig. 1) and same-day transfer to a PCI centre for such patients. In previous reports, we have communicated our appraisal of and thoughts on the early invasive strategy and same-day transfer recommendations in the 2015 NSTE-ACS Guidelines [15, 16]. Since the publication of these guidelines, three new RCTs have been published focusing on the timing of angiography within the routine invasive strategy.

For daily clinical practice, it is noteworthy to realise that the interpretation of trial data is limited by several factors. The first limitation is how the time to ICA is measured. In most trials, this is based on the time from randomisation and not the time from symptom onset or hospital admission. Second, treatment strategy trials comparing an early with a delayed treatment strategy use different timing definitions.

The EARLY trial was an open-label RCT that compared immediate (median time between randomisation and angiography 0 h) versus early angiography (median time 18 h) in 741 NSTE-ACS patients (median Global Registry of Acute Coronary Events (GRACE) risk score 121 vs 123). The primary endpoint rate (composite of cardiovascular death and recurrent ischaemic events at 1 month) was significantly lower in the immediate group (4.4% vs 21.3%, HR 0.20, *p* < 0.001). However, this difference was driven by a reduction in the number of symptoms of ischaemia with dynamic electrocardiogram changes (2.9% vs 19.8%, *p* < 0.001). No difference was observed in rate of cardiovascular death or MI.

Although smaller in size but quite similar in timing strategy, the OPTIMA‑2 trial did not show any benefit at 1 year follow-up of an immediate (< 3 h) ICA strategy compared with an early one (< 24 h). The trial was terminated early for futility after 249 NSTE-ACS patients were randomised [17].

The RIDDLE-NSTEMI study, a small RCT with 323 NSTEMI patients, compared an immediate (median 1.4 h) with a delayed ICA strategy (median 61.0 h). The rate of death, new MI or recurrent ischaemia was lower in the immediate-intervention group at both 30 days (6.8% vs 26.7%, *p* < 0.001) and 1 year (15.4% vs 33.1%, *p* < 0.001). This difference was mainly driven by a reduction in MI rate [18, 19].

In the VERDICT trial, 2147 patients with a clinical suspicion of NSTE-ACS were included [20]. The primary endpoint of all-cause death, non-fatal recurrent MI, hospital admission for refractory ischaemia or hospital admission for heart failure did not differ between the early invasive group (median 4.7 h) and the delayed invasive group (median 61.6 h). However, among patients with a GRACE risk score > 140, a reduction in the primary endpoint was observed (HR 0.81, 95% CI 0.67–1.01, *p*-value for interaction = 0.023); however, which separate endpoints drove this outcome difference were not reported.

#### Summary

Evidence for an early invasive strategy < 24 h in patients with NSTE-ACS is still weak in terms of hard clinical outcomes. However, the largest VERDICT and TIMACS trials demonstrated a benefit in the subgroup of patients with GRACE risk score > 140. In a recent evaluation in the Netherlands, around 40% of the NSTE-ACS patients were high-risk as defined by a GRACE risk score > 140 [21]. Other arguments in favour of the early invasive strategy include an earlier diagnosis with consequences for (antithrombotic) pharmacological treatment, less recurrent ischaemia, and shorter hospital stay.

Taking these considerations into account, including the abovementioned large proportion of high-risk patients (GRACE risk score > 140) in the recent Dutch evaluation, we recommend complying to the ESC Guidelines with regards to timing of angiography (preferably < 24 h). If this is not possible from a logistical perspective, a delayed invasive (ICA within 72 h) or selective invasive strategy (ICA if recurrent chest pain or positive non-invasive ischaemia testing before discharge, in case routine coronary angiography is not attractive) is an acceptable and safe alternative (Fig. 1).

### Invasive coronary angiography in resuscitated patients after cardiac arrest

The 2020 ESC Guidelines recommend to perform ICA < 24 h in patients presenting after resuscitation for cardiac arrest without ST-segment elevation or cardiogenic shock. However, this strategy is mainly derived from observational studies. On the other hand, in the recently published COACT trial, patients who had been successfully resuscitated after out-of-hospital cardiac arrest and had no signs of STEMI, a strategy of immediate angiography was not found to be better than a strategy of delayed angiography with respect to overall survival at 90 days [22]. Median time from randomisation to coronary angiography in the delayed angiography group was 119 h in this study. For this reason, the Dutch ACS working group does not recommend ICA < 24 h in the context of resuscitation after cardiac arrest and NSTE-ACS. It is justifiable to await neurological recovery before performing ICA.

## Transfer to PCI centre

The recommendation to transfer all high-risk NSTE-ACS patients to a PCI centre < 24 h is not based on scientific evidence. Therefore, we advise to consider this recommendation in the context of regional arrangements between PCI centres and referring hospitals. Possible considerations for performing ICA in a centre with PCI capabilities are to avoid two invasive procedures in the same patient and related potential complications. On the other hand, angiography in a non-PCI centre may relieve the burden on ambulance services, as a substantial number of NSTE-ACS patients do not undergo PCI. In addition, time between ICA at the referral centre and the PCI could enable improved pre-procedural preparation (antithrombotic treatment, renal protection) for those who do undergo PCI.

## Conclusion

In line with the 2020 ESC Guidelines for NSTE-ACS, the Dutch ACS working group and the Working Group of Interventional Cardiology endorse the deferral of pretreatment with P2Y_12_ receptor inhibitors until the diagnosis is confirmed by and the coronary anatomy is visualised with ICA within 24 h. In contrast to the 2020 ESC Guidelines, we cannot state a preference for prasugrel nor ticagrelor as first-choice therapy.

Second, we endorse the very high- and high-risk criteria for immediate and early invasive strategies. The preference to perform ICA < 24 h in the context of the Dutch situation (> 40% of NSTE-ACS patient population has a GRACE risk score > 140) is also recommended by the Dutch working groups. The Netherlands shows a well-organised and dense capacity of ICA laboratories. For this reason, if no very high- or high-risk criteria are met, the absence of evidence for improved prognosis in *routine* early invasive strategy justifies a delayed strategy (ICA < 72 h) as a safe alternative.

For medically managed patients with NSTE-ACS, the Dutch working groups recommend pretreatment with ticagrelor in those with a low bleeding risk and pretreatment with clopidogrel in patients > 70 years of age or in patients with a high bleeding risk.

In contrast to the 2020 ESC Guidelines, we do not recommend ICA < 24 h in resuscitated patients with NSTE-ACS, but instead to await neurological recovery.

Routine transfer to a PCI centre < 24 h is also not recommended, but instead needs to be evaluated in the context of regional arrangements between PCI centres and referring hospitals to avoid repeated invasive procedures, to relieve the burden on ambulance services and PCI centres and to improve patient preparation when the coronary anatomy is known.
